# News and Miscellany

**Published:** 1902-08

**Authors:** 


### News and Miscellany.

**A Young State Health Officer** for Texas was born to Doctor and Mrs. Geo. R. Tabor at Austin July 9th, ult.

**Dr. John Preston** of Lockhart, delivered a German woman of triplets recently; two boys and a girl. None of them got away.

**The Board of Health** of the province of Quebec has called upon Montreal to pass a by-law providing for compulsory vaccination.

**The New Jersey Legislature** has recently appropriated $50,000 to build and equip a sanitarium for the care of indigent consumptives.

**The Wistar Institute of Anatomy** of the University of Pennsylvania has received the bodies of two infants joined as were the famous Siamese twins. They had only one umbilical cord and one placenta, and were still-born.

**Wanted:** To purchase a location, or a partnership in a well established practice, by a regular physician of twenty years experience. Can give best reference. Must bear strict investigation. Give full particulars in first communication. Address, "X. Ray," care TEXAS MEDICAL JOURNAL, Austin, Texas.

---

**Constipation,** like the poor, is always with us. (This is *not* an editorial confession.)

Cascara is the "Balm in Gilead." S. & D.'s is the "balmiest." They talk about it in the RED BACK this month. Of course you've read it. You always read their ads. But may be you haven't sent for a sample or for their monograph.

---

**The President has** designated Col. R. M. O'Reilly to be surgeon general of the army, to succeed General Forwood, who will retire on September 7, next. Colonel O'Reilly will have until January, 1909, to serve as surgeon general. He was appointed from Pennsylvania as a medical cadet in 1864. He is a graduate of the medical department of the University of Pennsylvania.

---

**Centenary Hospital.**—The Faculty of the Barnes Medical College, at a cost of nearly $150,000, have completed the new "Centenary Hospital." The beautiful six-story building is in direct connection with college, corner of Garrison and Lawton avenues. It is modern, with capacity for one hundred and fifty patients, and strictly fire-proof throughout, and furnished with every modern hospital convenience. No expense has been spared to make it the best hospital in St. Louis.

---

**A Texas Boy Honored.**—Dr. Harry C. Loew, a native of Houston, Texas, a grandson of Dr. A. S. Wolff, the long time and able quarantine officer at Brownsville, Texas,—and the fourth generation of doctors—graduated M. D. from Columbia Medical College June 11, 1902. By competitive examination he won a position upon the Staff of Kings County Hospital, Brooklyn, where he is now on duty. The RED BACK congratulates "The Count," as I call grandpa Wolff.

---

**"Our Friend Dan'ls."**—This genius who runs the "Red Back," or better known as the *Texas Medical Journal*—even better known as "Dan'ls Texas Journal," announces that it is entering its eighteenth year of publication. Bro. Daniel is congratulated on having run successfully the brightest, cleanest, livest and Jim-Dandyest journal in the country for the past seventeen years, and he offers to keep the ball rolling as before, only a little better. Go on my brother, you are a brick, and may nothing ever impede your glorious progress is the hope of the *Southern Clinic.*

